# *IL13 *genetic polymorphisms, smoking, and eczema in women: a case-control study in Japan

**DOI:** 10.1186/1471-2350-12-142

**Published:** 2011-10-21

**Authors:** Yoshihiro Miyake, Keiko Tanaka, Masashi Arakawa

**Affiliations:** 1 Department of Preventive Medicine and Public Health, Faculty of Medicine, Fukuoka University, Fukuoka, Japan; 2 Field Science for Health and Recreation, Faculty of Tourism Sciences and Industrial Management, University of the Ryukyus, Okinawa, Japan

## Abstract

**Background:**

Several genetic association studies have examined the relationships between single nucleotide polymorphisms (SNPs) in the *IL13 *gene and eczema, and have provided contradictory results. We investigated the relationship between the *IL13 *SNPs rs1800925 and rs20541 and the risk of eczema in Japanese young adult women.

**Methods:**

Included were 188 cases who met the criteria of the International Study of Asthma and Allergies in Childhood (ISAAC) for eczema. Control subjects were 1,082 women without eczema according to the ISAAC criteria, who had not been diagnosed with atopic eczema by a doctor and who had no current asthma as defined by the European Community Respiratory Health Survey criteria. Adjustment was made for age, region of residence, number of children, smoking, and education.

**Results:**

The minor TT genotype of SNP rs1800925 was significantly associated with an increased risk of eczema in the co-dominant model: the adjusted odds ratio was 2.19 (95% confidence interval: 1.03-4.67). SNP rs20541 was not related to eczema. None of the haplotypes were significantly associated with eczema. Compared with women with the CC or CT genotype of SNP rs1800925 who had never smoked, those with the TT genotype who had ever smoked had a 2.85-fold increased risk of eczema, though the adjusted odds ratio was not statistically significant, and neither multiplicative nor additive interaction was statistically significant.

**Conclusions:**

Our findings suggest that the *IL13 *SNP rs1800925 is significantly associated with eczema in Japanese young adult women. We could not find evidence for an interaction between SNP rs1800925 and smoking with regard to eczema.

## Background

In Japan, atopic eczema is a major public health problem not only among children but also among adults. One study has reported atopic eczema prevalence values of 10.9% and 7.8% among Japanese women in their 20s and 30s, respectively [1]. The role of genetic and environmental risk factors in the development of atopic eczema has gained interest.

Interleukin (IL)-13 is a central mediator of Th2 immune responses [2]. IL-13 is expressed in acute and chronic lesions and in unaffected skin from patients with atopic eczema [3]. T cells and mast cells are important sources of IL-13 in skin lesions from patients with atopic eczema [4]. The percentage of CD4^+ ^IL-13^+ ^T cells has been shown to correlate significantly with the severity of atopic eczema in children [5]. High IL-13 production by phytohaemagglutinin- and house dust mite allergen Der p 1-stimulated cord blood mononuclear cells has been associated with the development of atopic eczema by the age of 3 years [6].

Several genetic association studies have examined relationships between single nucleotide polymorphisms (SNPs) in the *IL13 *gene and eczema, and have provided contradictory results [7-15]. Our recent study of Japanese children aged 3 years showed that the minor TT genotype of SNP rs1800925 (-1024C/T) and the minor AA genotype of SNP rs20541 (Arg110/130Glu) were significantly related to an increased risk of eczema and that perinatal smoking exposure did not interact with the 2 SNPs in the etiology of eczema [15]. These 2 SNPs are functional SNPs. Having established this, we wished to investigate these issues in Japanese adults using data from the Kyushu Okinawa Maternal and Child Health Study (KOMCHS).

## Methods

### Study Population

The KOMCHS is an ongoing prospective prebirth cohort study that investigates risk and preventive factors for maternal and child health problems such as allergic disorders. From April 2007 to March 2008, the KOMCHS requested that 131 obstetric hospitals in Fukuoka Prefecture, the largest prefecture on Kyushu Island in southern Japan, with a total population of approximately 5.04 million, provide as many pregnant women as possible with a set of leaflets explaining the KOMCHS, an application to participate in the study, and a self-addressed stamped envelope in which to return the application. From May 2007 to March 2008, the KOMCHS also requested that 40 obstetric hospitals in Okinawa Prefecture, one of the southernmost islands of Japan, with a total population of almost 1.37 million, provide as many pregnant women as possible with a comparable set of documents. Later, to increase the sample size, pregnant women living in 6 prefectures on Kyushu Island other than Fukuoka Prefecture, with a total population of approximately 8.22 million, were provided with comparable documents at 252 obstetric hospitals between August 2007 and March 2008. Pregnant women who intended to participate in the KOMCHS returned the application form to the data management center. By the end of the study, a total of 1757 pregnant women between the 5th and 39th week of pregnancy had given their fully informed consent in writing to participate in the KOMCHS and had completed the baseline survey. Around four months after delivery, 1492 women gave informed consent to genotyping. The ethics committee of the Faculty of Medicine, Fukuoka University, approved the KOMCHS.

### Selection of Cases and Controls

Women with eczema were identified based on the International Study of Asthma and Allergies in Childhood (ISAAC) Phase One questionnaire [16]. Affirmative answers to the following 3 questions were required: 'Have you ever had an itchy rash which was coming and going for at least 6 months?', 'Have you had this itchy rash at any time in the last 12 months?' and 'Has this itchy rash at any time affected any of the following places: the folds of the elbows, behind the knees, in front of the ankles, under the buttocks, or around the neck, ears, or eyes?' Eventually, 188 cases of eczema were identified.

Of the 1304 remaining participants who were therefore eligible to serve as controls, 173 women were excluded from the analysis because they answered 'yes' to the question: 'Have you ever been diagnosed by a physician as having atopic eczema?'. Sixty-six were excluded who provided a positive response to either of 2 situations: an asthma attack during the last 12 months or current use of asthma medication, with asthma as defined by the European Community Respiratory Health Survey [17]. Finally, 1 mother was excluded because of missing data on smoking. Because 18 mothers had both doctor-diagnosed atopic eczema and current asthma, 1082 control subjects remained for the final analysis.

### DNA Extraction and Genotyping

Research technicians or subjects themselves collected buccal specimens with BuccalAmp swabs (Epicenter BioTechnologies, Madison, WI, USA). Genomic DNA was extracted using a QIAmp DNA mini kit (Qiagen, Inc., Valencia, CA, USA). Two SNPs from the *IL13 *gene were used in this study: rs1800925 in the 5' promoter region and rs20541 in exon 4. Genotyping of the two *IL13 *polymorphisms was performed using pre-development TaqMan SNP Genotyping Assays (Applied Biosystems, Foster City, CA, USA).

### Statistical Analysis

Deviation from the Hardy-Weinberg equilibrium among control subjects was evaluated by the chi-square test. Linkage disequilibrium was investigated using Haploview software version 4.1 [18]. Logistic regression analysis was used to obtain the crude odds ratios (ORs) and 95% confidence intervals (CIs) for eczema relative to the SNPs under study. Multiple logistic regression analysis was used to adjust for age, region of residence, number of children, smoking, and education. The statistical power calculation was performed using QUANTO version 1.2 [19]. Haplotypes and their frequencies were inferred with the expectation maximization (EM) algorithm. For differences in haplotype frequency between the case and control groups, crude ORs and 95% CIs were estimated based on the frequency of each haplotype relative to all other haplotypes combined. The multiplicative interaction was tested using a term of the product of two variables in a multiple logistic regression model. Three measures were used to test the additive interaction [20]: 1) relative excess risk due to interaction (RERI), 2) attributable proportion due to interaction (AP), and 3) synergy index (S). RERI is the excess risk due to an interaction relative to the risk without exposure. AP refers to the attributable proportion of disease that is due to an interaction among individuals with both exposures. S is the excess risk from both exposures when there is an additive interaction, relative to the risk from both exposures without an interaction. RERI = 0, AP = 0, or S = 1 means no interaction or strict additivity; RERI > 0, AP > 0, or S > 1 means positive interaction or more than additivity; RERI < 0, AP < 0, or S < 1 means negative interaction or less than additivity [21]. If any of the null values (0 in RERI and AP or 1 in S) falls outside the 95% CI of its respective measurement, then the additive interaction is considered statistically significant. Details of the method for the calculation of the additive interaction have been described by Andersson et al. [20]. Excluding the calculation of linkage disequilibrium and statistical power calculation, all statistical analyses were performed using STATA/SE software version 12.0 (StataCorp, College Station, TX, USA).

## Results

Compared with control subjects, women with eczema were more likely to be younger (Table 1). There were no differences between eczema cases and control subjects with regard to region of residence, number of children, smoking, and education.

**Table 1:** Characteristics of the study population

	*n *(%) or mean (SD)		
	
Variable	Cases (*N *= 188)	Controls (*N *= 1,082)	*P *value*
Age, years	30.7 (4.6)	31.6 (4.2)	0.006
Region of residence (%)			0.50
Fukuoka Prefecture	112 (59.6)	610 (56.4)	
Other than Fukuoka Prefecture in Kyushu	61 (32.5)	357 (33.0)	
Okinawa Prefecture	15 (8.0)	115 (10.6)	
Having one or more children (%)	107 (56.9)	669 (61.8)	0.20
Having ever smoked (%)	63 (33.5)	336 (31.1)	0.50
Education (%)			0.77
< 13 years	44 (23.4)	228 (21.1)	
13-14 years	62 (33.0)	368 (34.0)	
≥ 15 years	82 (43.6)	486 (44.9)	

* χ^2 ^test or t test.

*IL13 *SNPs rs1800925 and rs20541 were tested for deviation from the Hardy-Weinberg equilibrium; there was no deviation among control subjects (p = 0.10 and 0.83, respectively). SNPs rs1800925 and rs20541 were in high linkage disequilibrium (*D*' = 0.84, *r*^2 ^= 0.37).

The frequency of the TT genotype of SNP rs1800925 was 5.3% in cases and 2.5% in control subjects (Table 2). Compared to a reference group of subjects with the CC genotype, women with the TT genotype had a significantly increased risk of eczema, whereas no association was found between the CT genotype and eczema in crude analysis. Adjustment for confounders under investigation did not appreciably change the results: the adjusted ORs for the CT and TT genotypes were 1.01 (95% CI: 0.71-1.42) and 2.19 (95% CI: 1.03-4.67), respectively. In the recessive model, the TT genotype was significantly positively associated with eczema: the adjusted OR was 2.19 (95% CI: 1.04-4.63); however, no significant relationship was observed in the additive or dominant model. We could not find a significant association between SNP rs20541 and eczema in any genetic model. With respect to SNP rs20541, the statistical power calculation revealed that, using our sample size, we could detect the gene-disease association for an OR of 1.397 with an accuracy of more than 80% under the log-additive model.

**Table 2:** Odds ratios and 95% confidence intervals for eczema according to *IL13 *polymorphisms in Japanese women

SNP	Model	Genotype	Cases *n *(%)	Controls *n *(%)	Crude OR (95% CI)	Adjusted OR (95% CI)*
rs1800925			(*N *= 188)	(*N *= 1,082)		
	Co-dominant	CC	122 (64.9)	717 (66.3)	1.00	1.00
		CT	56 (29.8)	338 (31.2)	0.97 (0.69-1.37)	1.01 (0.71-1.42)
		TT	10 (5.3)	27 (2.5)	2.18 (1.03-4.61)	2.19 (1.03-4.67)
	Additive				1.15 (0.87-1.52)	1.18 (0.89-1.56)
	Dominant	CC	122 (64.9)	717 (66.3)	1.00	1.00
		CT + TT	66 (35.1)	365 (33.7)	1.06 (0.77-1.47)	1.10 (0.79-1.52)
	Recessive	CC + CT	178 (94.7)	1,055 (97.5)	1.00	1.00
		TT	10 (5.3)	27 (2.5)	2.20 (1.04-4.61)	2.19 (1.04-4.63)
rs20541			(*N *= 188)	(*N *= 1,081)		
	Co-dominant	GG	85 (45.2)	536 (49.6)	1.00	1.00
		GA	82 (43.6)	448 (41.4)	1.15 (0.83-1.60)	1.16 (0.84-1.62)
		AA	21 (11.2)	97 (9.0)	1.37 (0.81-2.31)	1.44 (0.85-2.44)
	Additive				1.16 (0.92-1.47)	1.19 (0.94-1.50)
	Dominant	GG	85 (45.2)	536 (49.6)	1.00	1.00
		GA + AA	103 (54.8)	545 (50.4)	1.19 (0.87-1.63)	1.21 (0.89-1.66)
	Recessive	GG + GA	167 (88.8)	984 (91.0)	1.00	1.00
		AA	21 (11.2)	97 (9.0)	1.28 (0.77-2.10)	1.34 (0.81-2.22)

* Adjusted for age, region of residence, number of children, smoking, and education.

Four haplotypes were constructed, but none of these were significantly associated with the risk of eczema (Table 3).

**Table 3:** Haplotype analysis of rs1800925 and rs20541 in relation to eczema in Japanese women

Haplotype	Haplotype structure		Frequency *n *(%)		
	
number	rs1800925	rs20541	Cases (2*N *= 376)	Controls (2*N *= 2,164)	Crude OR (95% CI)*
1	C	G	249 (66.2)	1,475(68.2)	0.92 (0.72-1.16)
2	C	A	51 (13.6)	297 (13.7)	0.99 (0.70-1.37)
3	T	G	3 (0.8)	47 (2.2)	0.36 (0.07-1.14)
4	T	A	73 (19.4)	345 (15.9)	1.27 (0.95-1.69)

* Crude OR for each haplotype is relative to all other haplotypes combined.

Compared with women with the CC or CT genotype of SNP rs1800925 who had never smoked, those with the TT genotype who had ever smoked had a 2.85-fold increased risk of eczema, although the adjusted OR was not statistically significant, and neither multiplicative nor additive interaction was statistically significant (Table 4).

**Table 4:** Interaction between *IL13 *rs1800925 polymorphism and smoking

	Smoking status			
	
	No		Yes	
	
SNP	No. of cases/controls	Adjusted OR (95% CI)*	No. of cases/controls	Adjusted OR (95% CI)*
CC + CT	119/727	1.00	59/328	1.06 (0.75-1.50)
TT	6/19	1.96 (0.76-5.03)	4/8	2.85 (0.83-9.73)
*P *for multiplicative interaction = 0.69				
Measures of additive interaction^†^				
Relative excess risk due to interaction (RERI) = 0.83 (95% CI: -3.08-4.74)				
Attributable proportion due to interaction (AP) = 0.29 (95% CI: -0.78-1.37)				
Synergy index (S) = 1.82 (95% CI: 0.13-24.85)				

* Adjusted for age, region of residence, number of children, and education.

^† ^Statistically significant with the 95% CI of RERI > 0, the 95% CI of AP > 0, or the 95% CI of S > 1, indicating additive interaction.

The results of a sensitivity analysis restricted to cases without current asthma as defined by the European Community Respiratory Health Survey (n = 165) were similar to those in the overall analysis: the adjusted OR was 2.51 (95% CI: 1.18-5.36) for the TT genotype of SNP rs1800925 and 1.33 (95% CI: 0.75-2.36) for the AA genotype of SNP rs20541 in the co-dominant model.

## Discussion

Our previous study of Japanese children aged 3 years observed that both *IL13 *SNPs rs1800925 and rs20541 were significantly associated with the risk of eczema [15]. Interestingly, in that study, after mutual adjustment for SNPs rs1800925 and rs20541, the positive relationship between SNP rs1800925 and eczema remained statistically significant, whereas the positive association between SNP rs20541 and eczema completely disappeared [15]. The current results are in partial agreement with our previous findings. Another Japanese case-control study, which included patients with atopic eczema aged 11-61 years, showed a significant association between SNP rs20541 and atopic eczema [8]. Of the genetic association studies conducted in countries other than Japan, to our knowledge, only 1 case-control study of Danish subjects aged 17-66 years found a significant relationship between SNP rs1800925 and atopic eczema [9]. In contrast, a null association between SNP rs1800925 and eczema was observed in studies of Canadian [10], US [11], and UK [13] children as well as in a study of children aged 8-12 years from 13 countries [14] and a study of Taiwanese young adults [12]. Two studies of German [7] and Canadian [10] children found a significant association between SNP rs20541 and eczema, whereas a relationship between SNP rs20541 and eczema was ruled out in 2 studies of US [11] and UK [13] children and in a study of Taiwanese adults [12]. These discrepancies may be at least partly explained by differences in genetic background of the populations examined, definitions of eczema, and statistical power.

In a study in the Netherlands, subjects with the TT genotype of SNP rs1800925 exhibited significantly weaker relative inhibition of IL-13 production upon additional stimulation with anti-CD2 compared to those with the CC or CT genotype [22]. Cameron and colleagues have shown that the T allele of SNP rs1800925 enhanced *IL13 *promoter activity in primary human and murine CD4^+ ^Th2 lymphocytes through Yin-Yang 1-dependent attenuation of STAT6-mediated promoter repression and that mitogen-activated peripheral blood mononuclear cells from pregnant women with the TT genotype of SNP rs1800925 secreted significantly higher levels of IL-13 compared with those from pregnant women with the CC or CT genotype [23]. A laboratory study has shown that the IL-13 Glu110 variant is significantly more active than wild-type IL-13 in inducing STAT6 phosphorylation and CD23 expression in primary human monocytes as well as hydrocortisone-dependent IgE switching in human B cells, and that the IL-13 Glu110 variant was neutralized less effectively than wild-type IL-13 by a soluble form of IL-13Rα2 [24].

A study in Germany reported that secondhand smoke exposure increased IL-13 levels in children with asthma [25]. In a study in the UK, a significant interaction was found between SNP rs1800925 and smoking with respect to the risk of allergy [26]. In our previous study, however, there were no interactions between perinatal smoking exposure and SNPs rs1800925 and rs20541 in the etiology of childhood eczema [15]. In the current study, no significant interaction was observed between SNP rs1800925 and smoking with respect to eczema.

The current study offers two main methodological advantages: study subjects were homogeneous in that they were all pregnant women, and adjustment was made for several confounders. Nevertheless, we could not rule out residual confounding because of other unknown variables. It is also subject to several limitations, however: first, the participation rate cannot be calculated because the exact number of eligible pregnant women who were provided with the abovementioned KOMCHS documents is not available. In addition, we were not able to assess differences between participants and non-participants, because information on personal characteristics such as age, socioeconomic status, and a history of allergic disorders among the non-participants was not available. Our subjects were probably not a representative sample of Japanese women in the general population; it can be shown, for example, that educational levels were higher in the current study population than in the general population. According to the 2000 population census of Japan, the proportions of women aged 30 to 34 years in Fukuoka Prefecture with years of education of < 13, 13-14, ≥ 15, and unknown were 52.0%, 31.5%, 11.8%, and 4.8%, respectively [27]. The corresponding figures for the current study in the control group were 21.1%, 34.0%, 44.9%, and 0.0%, respectively. Thus the present population might have had greater awareness concerning health-related matters compared to the general population. Regardless, the distribution of the 2 SNPs under study in the control group was in agreement with the Hardy-Weinberg equilibrium.

The definition of eczema was based on the questions in the ISAAC questionnaire, although validation tests of such questions have not been performed for Japanese young adults. Data on serum-specific IgE levels or skin prick tests were not available. The possibility of non-differential outcome misclassification might have biased the magnitude of the observed associations toward the null.

Control subjects were women without eczema as defined by the ISAAC criteria who had not been diagnosed by a doctor with atopic eczema and had no current asthma as defined by the European Community Respiratory Health Survey. To minimize the effect of allergic sensitization based on asthma in our cases, we performed a sensitivity analysis excluding cases with current asthma: the results of the sensitivity analysis were similar to those in the overall analysis.

The number of cases was rather small for a valid genetic association study, but a significant association with SNP rs1800925 was nevertheless detected.

Correction for multiple testing was not performed in the current study. As this is a hypothesis testing study and part of the current findings is a replication of previously published results, we think that correction for multiple testing would cause us to underestimate our results.

## Conclusions

The current study suggests that *IL13 *SNP rs1800925, but not rs20541, is significantly associated with the risk of eczema in Japanese young adult women. To our knowledge, this is the first study to find a significant association between SNP rs1800925 and eczema in non-Western adult populations. We could not provide evidence for an interaction between SNP rs1800925 and smoking with respect to eczema. Additional studies with a greater number of cases are required to confirm these findings.

## Abbreviations

CI: Confidence interval; ISAAC: International Study of Asthma and Allergies in Childhood; KOMCHS: Kyushu Okinawa Maternal and Child Health Study; OR: Odds ratio; SNP: single nucleotide polymorphism.

## Competing interests

The authors declare that they have no competing interests.

## Authors' contributions

YM, KT, and MA contributed to the study concept and design and the acquisition of data. YM was responsible for the analysis and interpretation of data and the drafting of the manuscript. All authors participated in critically revising the manuscript and approved the final version of the manuscript.

## Pre-publication history

The pre-publication history for this paper can be accessed here:

http://www.biomedcentral.com/1471-2350/12/142/prepub
